# Fertility detection of unincubated chicken eggs by hyperspectral transmission imaging in the Vis-SWNIR region

**DOI:** 10.1038/s41598-024-51874-2

**Published:** 2024-01-14

**Authors:** Mahdi Ghaderi, Seyed Ahmad Mireei, Aminollah Masoumi, Mohammad Sedghi, Majid Nazeri

**Affiliations:** 1 Department of Biosystems Engineering, College of Agriculture, Isfahan University of Technology, Isfahan, 84156-83111 Iran; 2 Department of Animal Science, College of Agriculture, Isfahan University of Technology, Isfahan, 84156-83111 Iran; 3 Department of Laser and Photonics, Faculty of Physics, University of Kashan, Kashan, 87317-53153 Iran

**Keywords:** Applied optics, Statistics

## Abstract

Detection of infertile eggs prior to incubation can lead to an increase in the hatchability rate and prevent the wastage of billions of non-fertile eggs ended up by failed incubation. In this study, the feasibility of a line-scan hyperspectral imaging system in the visible and short-wavelength near-infrared region was assessed for early detection of non-fertile eggs on day 0 before incubation. A total of 227 white-shell eggs including 131 fertile and 96 infertile eggs were collected from a flock with similar conditions in terms of hen age, feeding, and management. Hyperspectral images of eggs were captured on day 0 before incubation in a transmittance mode of illumination and then the eggs were incubated in a commercial incubator. The edge detection method was used to segment the egg, including both the white and yolk, from the background, and the image spectral information was extracted from the egg region. After applying various pretreatment methods, different classifiers including soft independent modeling of class analogy (SIMCA), linear discriminant analysis (LDA), quadratic discriminant analysis (QDA), and artificial neural networks (ANN) classifiers were utilized to extract the predictive models. Following the acceptable results of SIMCA analysis accomplished by 1st derivative pretreatment (accuracy of 86.67%), the discrimination power plot was used to select the most informative wavebands. The results showed that by using fewer variables in effective wavebands better performance (precision and accuracy of 92.59% and 93.33%, respectively) could be obtained in comparison with the ANN classifier based on the whole spectral data (precision and accuracy of 89.29% and 91.11%, respectively). This study revealed the potential application of hyperspectral transmittance imaging in the Vis-SWNIR region to discern the fertile and infertile eggs before starting the incubation process.

## Introduction

For poultry farm owners globally, detecting infertile eggs before incubation is an essential economic concern. Since the embryo at the initial stages of development is too small, fertility detection cannot be carried out by the traditional candling method. On the other hand, the hatchery statistics indicate that about 7–8% of the total number of eggs put into incubation remain unhatched, despite they should have been fertilized^1^. Therefore, commercial poultry hatcheries are incubating billions of eggs per year worldwide that are not supposed to hatch. In the long run, the main problem in the incubation of such unproductive infertile eggs is ending up the billions of eggs that could have been used for human consumption. Not only that, loss of energy due to the inefficient use of incubator space, increasing handling costs and decreasing hatchery production, and the risk of contamination of the whole eggs set by exploder eggs are considered as the other bottlenecks in incubating the non-fertile eggs^2–4^. Therefore, developing a non-destructive and more targeted method for early egg fertility identification, preferably before being passed for incubation, can improve the efficiency of the hatchery industry and promise huge economic returns.

Different methods have been introduced so far to separate unfertilized eggs, most of which are applicable in 2–5 days after incubation^3^. Conventional candling is the most popular method to assess flock fertility, usually performed 5–10 days after incubation. This method is not only slow and cumbersome but also approximately 5% of the entire egg set is randomly investigated while the remaining 95% have the chance of infertility^4^. Other approaches were presented to indirectly monitor the fertility of chicken eggs. In the best conditions, acceptable results for infertility detection were obtained by machine vision after 3 days^5^, thermal imaging after 14 days^6^, temperature sensors after 21 days^7^ of incubation, and visible and short-wavelength near-infrared (Vis-SWNIR) transmittance spectroscopy^8^ and NIR hyperspectral imaging in the day before incubation or day 0^2^.

Among these methods, the hyperspectral imaging (HSI) technique has recently been applied to detect egg fertility and embryo development^1,2,9^. In general, hyperspectral imaging is a type of spectral imaging that combines spectral data from a part of the electromagnetic spectrum with spatial information from a targeted sample. The main purpose of spectral imaging is to obtain spectral content or signature for every single pixel of the image^10^. The spectral signature is unique to different materials, such as each human fingerprint, and as a result, by obtaining this signature, it is possible to identify the amount and spatial distribution of different materials^11^.

More specifically, hyperspectral transmission imaging in the spectral range of Vis-SWNIR has been used to detect developing eggs with accuracies of 96%, 92%, 100%, and 100% on days 0, 1, 2, and 3 after incubation, respectively^12^. Afterward, in addition to recognizing the infertile eggs after starting the incubation process, Smith, et al.^13^ tried to detect the infertile eggs on day 0 before incubation. The results presented overall accuracy as 71% on day 0 before incubation, 63% on day 1, 65% on day 2, and 83% on day 3 after incubation. Zhihui, et al.^14^ utilized the entire spectral data within the wavelength range of 400–760 nm to detect fertile hatching eggs before incubation, achieving the highest accuracy of 93% through the application of a support vector machine classifier.

The most notable breakthrough in unraveling this problem was made by Liu and Ngadi^2^ who reported the detection of infertile eggs on day 0 before incubation, however, thanks to a relatively more expensive HSI system with the NIR spectral range (900–1700 nm). The best overall classification accuracies obtained were 100% on day 0, 79% on day 1, 74% on day 2, 82% on day 3, and 84% on day 4.

Despite the ability to detect fertility and monitor embryo development, no promising study, to the best of our knowledge, has been found on extracting effective wavelengths using the HSI systems for diagnosing chicken egg fertility on day 0 before incubation.

After collecting the hyperspectral images, it is necessary to extract the desired spatial and spectral features from the images. However, due to the small size of the egg embryo on day 0 and limitations in spatial resolution of hyperspectral cameras, it is difficult to visualize the embryo in hyperspectral images. Therefore, it seems that the spectral analysis in hyperspectral images is superior to spatial analysis, especially when other studies confirmed that the transmission spectra are affected by the presence of the embryo in the egg, causing some absorptions^3,15^.

In spectral analysis, choosing effective wavelengths is a critical step. Opting for the right wavelengths can decrease the dimensionality and complexity of data, ultimately enhancing the predictive capability of the model. The use of effective wavelengths not only reduces analysis time but is also advantageous for implementing the model in an online multispectral imaging system^16^. Moreover, given the vast amount of information in hyperspectral images, it becomes crucial to select the most appropriate classification method for discriminating the desired classes.

Different classification and wavelength selection approaches have been reported in the literature related to spectral analysis and they resulted in various prediction powers. For example, principal components analysis (PCA) in fertility detection by HSI^1,2^, naive Bayes classifier^3^, and linear discriminant analysis and support vector machine^15^ in fertility detection of eggs using Vis-SWNIR transmittance spectroscopy, and receiver operating characteristic (ROC) analysis in the wavelength difference of reflectance spectra in bruise detection in apple^16^, were examined.

In this paper, soft independent modeling of class analogy (SIMCA) was first utilized to investigate the feasibility of Vis-SWNIR spectral data based on the HSI technique for detecting non-fertile eggs. Afterward, the wavelength variables with strong discriminatory powers were retained, while the weaker wavelengths were excluded from the data set. The ability of the selected wavebands was checked after importing the raw and processed spectral data to the nonlinear artificial neural network (ANN) classifier.

The objectives of this study were (1) to evaluate the feasibility of hyperspectral transmission imaging in the spectral range of Vis-SWNIR to detect the non-fertile eggs before starting the incubation process, and (2) to determine and compare the potential of different machine learning tools in classifying the fertile and infertile eggs, and (3) selecting the most informative wavebands to develop the predictive models for detecting fertility of unincubated chicken egg. Enhancing the accuracy of fertility detection before the incubation process, through the use of a more cost-effective hyperspectral camera, the selection of the most informative wavelengths and development of the predictive model based on the discrete number of wavelengths, and the comparison of the performance of various machine learning techniques, can be considered the novel aspects of the current study.

## Materials and methods

### Sample preparation

A total of 227 clean, white-shell, fresh, unwashed eggs were prepared including 131 fertile and 96 infertile eggs. The eggs were collected from a flock of 60 Leghorn laying breeder hens (Hy-Line W-36). The birds were purchased from a commercial laying breeder farm and kept in the poultry farm belonging to the Isfahan University of Technology. Then, the hens were randomly distributed in two sub-flocks of 30 laying breeder hens in each. While the first sub-flock was raised without the rooster to produce non-fertile eggs, 4 roosters were added to the second sub-flock to create as much fertility as possible. All the sub-flocks were fed a similar and standard diet, therefore, the collected fertile and infertile eggs were prepared with similar conditions in terms of hen age, feeding program, and management. These conditions could be helpful to minimize errors and make the presence of the embryo the most important factor in providing the differences between the two groups of samples. In other words, the effect of other influential factors such as variations in egg-shell thickness and internal composition arising from the hen age and diet was diminished remarkably.

The egg samples were collected daily from both sub-flocks, then numbered and weighed and their dimensions were measured. After acquiring the hyperspectral images, the eggs were immediately incubated in a commercial incubator (Jam Toyor-504, Iran) under the standard conditions (temperature of 37.5 °C and relative humidity of 60%, turned every hour)^2^. After 5 days of incubation, eggs were candled and broken out to determine their fertility status. The infertile eggs were distinguished and related hyperspectral data were transferred to the proper sample group.

### Hyperspectral imaging system

**Figure 1:**
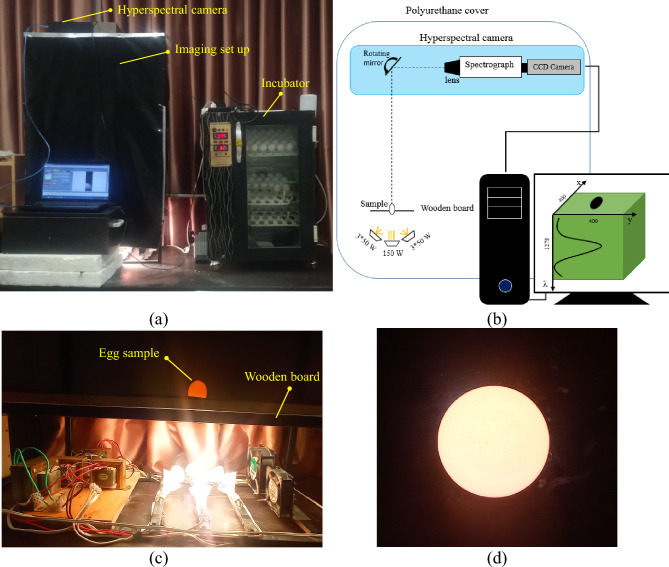
Hyperspectral imaging system for acquiring the egg pictures in full-transmittance mode (**a**) system set-up with main and secondary incubators, (**b**) The schematic of the hyperspectral imaging system, (**c**) light sources, and (**d**) the RGB image of the sample after illumination.

A line scanning visible and near-infrared (Vis–NIR) HSI system (model V1001, OPTC, Iran) was utilized to acquire spectral images in the full-transmittance mode in the range of 400–1000 nm and average optical spectral resolution of 2 nm (Fig. 1). The mirror of HSI camera was connected to a stepper motor that could take both spectral and spatial information of the illuminated egg without moving the sample (Fig. 1b). The exposure time was set at 100 ms after trial and error to reach the maximum possible signal-to-noise in the spectral data. To cover the entire width of the egg and achieve maximum spatial resolution, the number of scans was set at 400 with a distance of 100 cm between the camera and the sample. The HSI set-up was covered with a polyurethane cover to prevent ambient light from entering the imaging chamber (Fig. 1a).

The egg sample was vertically placed in a 5 cm diameter hole drilled in a wooden board between the camera and the light sources (Fig. 1c). By this arrangement, transmitted light passed through the egg could enter the camera. The light sources comprised one 150W lamp (at the center) and six 50W lamps (arranged around the center) of tungsten halogen, with color temperatures of 3270 K and 3200 K, respectively. These lamps were positioned beneath the sample, as shown in Fig. 1b. Two fans were used in the light exposure chamber with the direction of blowing from outside to inside (Fig. 1c). In addition to cooling the light sources and preventing the overheating of the egg samples, the blowing of warm air under the eggs could provide the heat required for the embryo’s survival while capturing the hyperspectral images.

The HSI images of samples were originally saved in the raw format, comprising 1279 images in λ direction as spectral dimension and 400 × 400 two spatial dimensions. Therefore, the original output hypercube had dimensions of 400 rows × 400 columns × 1279 bands. However, due to the absorption of eggshell^8^ and the decrease in the intensity of light sources in wavelengths below 500 nm, as well as the low signal-to-noise ratio of the camera’s detector in wavelengths above 950 nm, the transmission spectral data from 500–950 nm was utilized for predicting the fertility of chicken eggs before incubation. To enhance the signal-to-noise ratio, the averaging method was applied in the selected wavelength region, related to the dispersion of wavelength isolator in different wavelengths. This resulted in the final hypercube of 400 rows × 400 columns × 256 bands.

For spectral calibration, a reference image was captured by allowing light to pass through the camera lens while adjusting the exposure time to the minimum^2^. The dark image was obtained with an exposure time of 100 ms by turning off the light sources and covering the camera with its cap.

### Image processing and spectra extraction

In dealing with fertilized eggs on day 0, it should be noted that the embryo has not developed enough to be easily recognized without the egg breaking. The blastoderm, a single layer of embryonic epithelial tissue, in a fertilized egg is a symmetrical circular ring with approximately 3–4 mm in diameter, while the blastodisc in unfertilized eggs can be seen as an asymmetrical solid spot with a smaller diameter of about 2.5 mm. Furthermore, blastoderm in fertile eggs has a lower density of Area Pellucida (AP) and a higher density of Area Opaca (AO) regions around its perimeter^4^. In addition to changes in the density of AP and AO, molecular alterations also occur in fertilized eggs, offering an alternative indirect method for detecting unincubated fertile and infertile eggs. For instance, Padliya, et al.^17^ observed that 9 proteins increased in abundance in fertilized egg yolk compared to unfertilized egg yolk, while 9 proteins decreased in abundance in fertilized egg yolk relative to unfertilized egg yolk. Qiu, et al.^18^ and Qiu, et al.^19^ investigated alterations in egg white protein and the original albumen differences, revealing more than tenfold differences (P < 0.01) in abundance between fresh unfertilized and fertilized chicken egg whites. Whereas the small variation in size of blastoderm and blastodisc along with limitations in spatial resolution of hyperspectral images confine spatial analysis of fertilized unincubated eggs, the changes in the density of AP and AO and the protein content of egg yolk can make the spectral analysis a more adequate approach toward differentiating between fertilized and unfertilized eggs before incubation.

**Figure 2:**
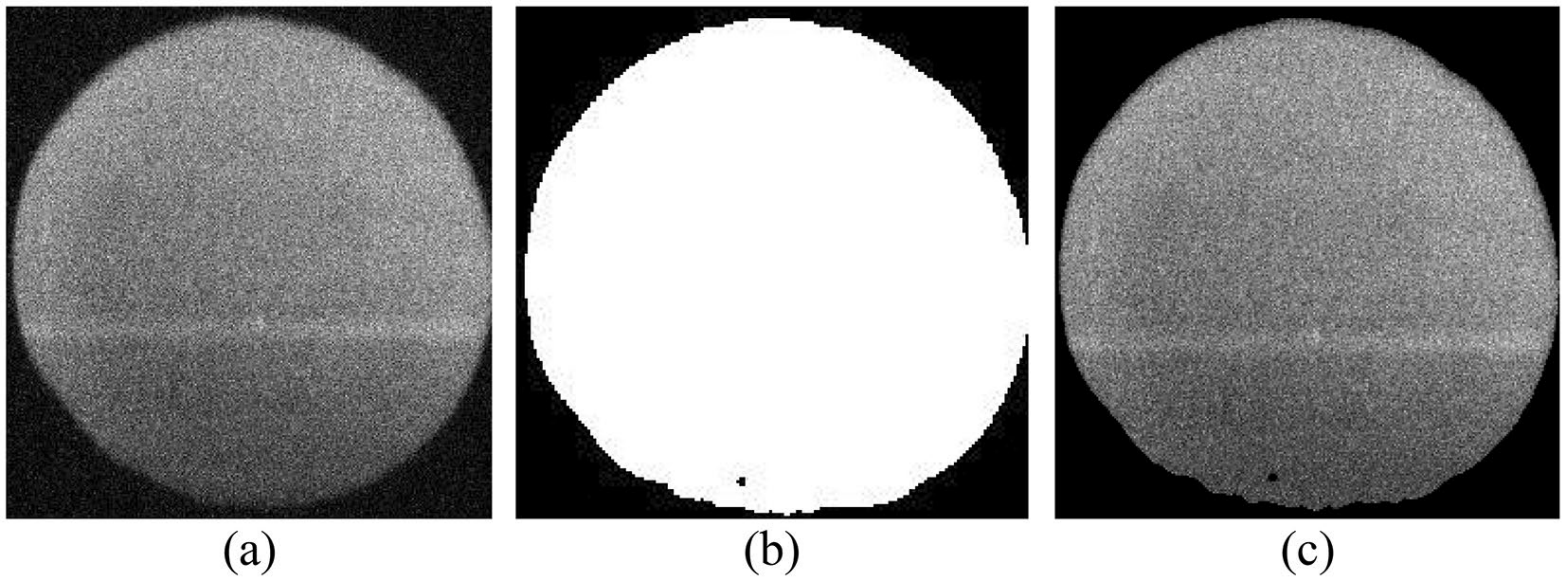
Background removal operations (**a**) the 630 nm raw image, (**b**) generated binary image, and (**c**) the 630 nm image without the background.

To speed up spectral processing and eliminate irrelevant information, the region of interest (ROI) of hyperspectral images should be confined to the egg region. Based on studies that emphasize the molecular differences between fertilized and unfertilized eggs, particularly the changes in protein content in yolk^17^ and albumen^18,19^, this research utilized the entire egg area as the ROI for subsequent image processing analysis. For this purpose, the best spectral image with the most contrast between the egg and the background was selected by visual investigation. This image was found around the wavelength of 630 nm (Fig. 2a) and was then used to perform background removal operations. The edge detection method was applied to segment the image into the egg and background and the binary image was produced (Zero code for the background and code one for the egg) (Fig. 2b). By performing the logical multiplication operation of the binary image on the other wavelength images, the hyperspectral image without background was obtained. This operation was performed on all acquired hyperspectral images.

In the next step, the average intensity of spectral data obtained from each image (*T*_*s*_) was calculated from the processed images. The relative transmittance spectrum of the samples (*T*_*rel*_) was then calculated to eliminate the interference by the optical system. This operation was done after collecting the dark and reference images. The intensity of dark (*T*_*d*_) and reference (*T*_*r*_) spectra were calculated by averaging the spectral data in the related images. Then, Eq. (1) was used to calculate the relative spectrum of the samples:

1 $$T_{rel} = \frac{{T_{s} /e_{s} - T_{d} /e_{s} }}{{T_{r} /e_{r} - T_{d} /e_{s} }} \times 100$$

where *e*_*s*_ and *e*_*r*_ are the exposure times of the camera while capturing the sample and reference images, respectively. Image processing and feature extraction approaches were all carried out using “MATLAB” V2014a (The MathWorks, Natick, USA).

### Spectral data analysis

**Figure 3:**
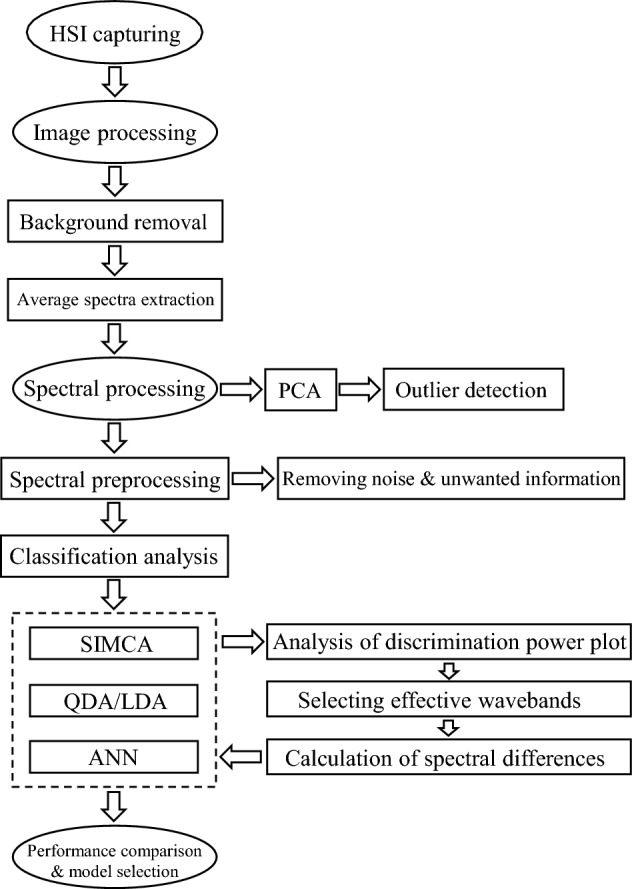
The proposed framework for detecting the fertile and infertile eggs and selecting the most relevant features.

The proposed framework for detecting the fertile and infertile eggs and selecting the most informative wavelength variables is shown in Fig. 3. After extracting the sample spectra, spectral preprocessing was performed to eliminate any undesired and irrelevant information due to the various factors such as light scattering effects and detector anomalies and highlight the differences between spectra for further analysis. The Savitsky-Golay smoothing method was first used to remove noise from the spectroscopy machine. In addition, the intensity of the radiation had a great effect on hyperspectral images. Therefore, methods such as standard normal variate (SNV) and normalization were used to minimize the effect of light intensity^20^. Other pretreatments such as baseline correction and multiplicative scatter correction (MSC) were also implemented to resolve the baseline changes and additive and/or multiplicative effects in spectral data, respectively. Finally, Savitzky-Golay first and second derivatives were used to correct the overlapping peaks and baseline variations, leading to broad bands being suppressed to sharp bands^21^.

Moreover, the principal components analysis (PCA) was used to review the spectral data and remove the outliers. The samples positioned outside the Hoteling T^2^ ellipse (with the 95% confidential level) were recognized as potential outliers and eliminated from the data set. Then, the data set was randomly disparted into two subsets, containing the calibration subset (80% of total samples) used for building the models, and an independent test subset (20% of remaining samples) applied to assess the ability of the models in prediction.

Generally, model building and feature selection approaches carried out in this study comprised two main steps. In the first step, the ability of different linear and nonlinear classifiers to discriminate the fertile and infertile classes was evaluated using all of the wavelength variables in the Vis-SWNIR region. In the next step, the most effective wavelength variables were selected by analysis of classifiers to reach a discrete number of wavelengths, suitable for multispectral imaging systems (Fig. 3).

#### Classification by whole spectral data

The machine learning tools used in this study included soft independent modeling of class analogy (SIMCA), linear discriminant analysis (LDA), quadratic discriminant analysis (QDA), and nonlinear artificial neural networks (ANN). These approaches are described briefly below, with key references cited for more details.

SIMCA is a supervised classifier that describes each class individually in PCA sub-models. The constructed sub-models are then used to evaluate the new sample’s belonging to the class. The result of SIMCA analysis is also very useful for evaluating the discrimination power of each wavelength variable. The discrimination power plot obtained from SIMCA analysis shows us which wavelengths are most effective for discerning between two classes. The variables that resulted in a discrimination power of more than 3 can be regarded as useful in distinguishing between two classes^22^.

LDA is another supervised pattern recognition method that is used to find the linear combination of features to separate two or more classes. It describes a linear separating hyperplane by calculating the linear discriminant functions. The linear functions are used to determine to which class an unknown sample belongs. In this method, the maximum ratio of the variance between the classes to the variance within the class is obtained in the direction of the normal vector of the separating hyperplane^23^.

QDA is a more general version of the LDA that classifies observations from different multivariate normal populations. While LDA calculates the Mahalanobis distance from a common covariance matrix considered for all classes, QDA derives the Mahalanobis distance from class-specific covariance matrices^24^. The QDA models used quadric surfaces to separate two or more classes based on Gaussian distribution. Then it used posterior distributions to guess the class for a given test sample. The Gaussian parameters are estimated based on maximum likelihood^25^. QDA is especially proposed when there is any conjecture about the gross violation from the homogeneity of within-classes variance.

In ANN analysis, multi-layer, feed-forward networks with the back-propagation (BP) learning algorithm were used for detecting egg fertility. The constructed ANN consisted of one input layer to transfer the spectral data processed by the best pretreatment into the network, one hidden layer, and one output layer with two nodes for fertile and infertile classes. The optimum number of nods in the hidden layer (NHL) was achieved after a trial and error procedure. For each number of NHL, three networks were developed and their average accuracy was calculated. The plot of average accuracy versus the number of NHL was used to find the optimum NHL and hence the best topology of networks.

After finding the best ANN model based on total spectral data, sensitivity analysis was performed to identify the most important input variables or wavelengths in explaining variances in the model output. Generally, for a trained network with specific parameters for each input variable, sensitivity analysis determines the effect of varying the parameters of the network for each variable on the overall network fit^26^.

The statistical parameters used to evaluate the classifiers comprised sensitivity (Sen.), specificity (Spe.), precision (Precis.), and accuracy (Acc.) defined by Eqs. (2) to (5), respectively^27^:

2 $$Sen = \frac{TP}{{TP + FN}}$$

3 $$Spe. = \frac{TN}{{FP + TN}}$$

4 $$Precis. = \frac{TP}{{TP + FP}}$$

5 $$Acc. = \frac{TP + TN}{{TP + FN + FP + TN}}$$

where TP (or true positive prediction) and TN (or true negative prediction) are the numbers of fertile and infertile eggs, respectively, that were correctly classified, and FP (or false positive prediction) and FN (or false negative prediction) are the numbers of infertile and fertile eggs that were misclassified as fertile and infertile, respectively.

Outlier detection, spectral pre-processing, PCA, and SIMCA, LDA, and QDA classifications were all performed using a statistical software package of ‘The Unscrambler’ V10.4 (CAMO AS, Trondheim, Norway). While, the ANN analysis was carried out by the neural networks package of ‘STATISTICA’ V12 (StatSoft, Inc., CA, USA).

#### Selecting the effective wavebands

Due to the great number of spectral bands in the hyperspectral images, the analysis of data is relatively slow. Therefore, multispectral imaging systems are suggested that work based on a certain number of wavelengths. These wavelengths should be the most informative ones, determined by analysis of hyperspectral images and successful predictive models.

Following the acceptable results of the SIMCA classifier accomplished by 1st derivative pretreatment, in the next step, the discrimination power plot was used to select the most influential wavebands. The effectiveness of selected wavebands in detecting fertility was evaluated by employing them as input for the ANN models. However, since these regions were discontinuous, the 1st derivative operation could not be carried out via common algorithms. Therefore, two approaches were selected and tested to overcome this problem. First, the raw spectral data of interested wavebands were used as the input to the ANN classifier, assuming that the preprocessing operation can be done by adjusting the weights in the nonlinear ANN method. In the second approach, the spectral difference between all possible pairs of wavelengths in the obtained effective wavebands was calculated to substitute the 1st derivative of transmission spectra^16^. The transmission difference values (*T*(λ_1_) − *T*(λ_2_)) were then used as the input of the ANN. Finally, the performance of constructed ANNs was compared and the best classifier based on the discrete number of wavelengths was presented.

### Ethics approval

Animal experiments were conducted in accordance with the Guiding Principles for the Care and Use of Research Animals at Isfahan University of Technology. The protocol and methods received approval from the Committee on Animal Experiment Ethics at Isfahan University of Technology (No. 390132). The study adhered to the ARRIVE guidelines for reporting animal research, experimental design, and data reporting^28^.

## Results and discussion

### Overview of spectral data

**Figure 4:**
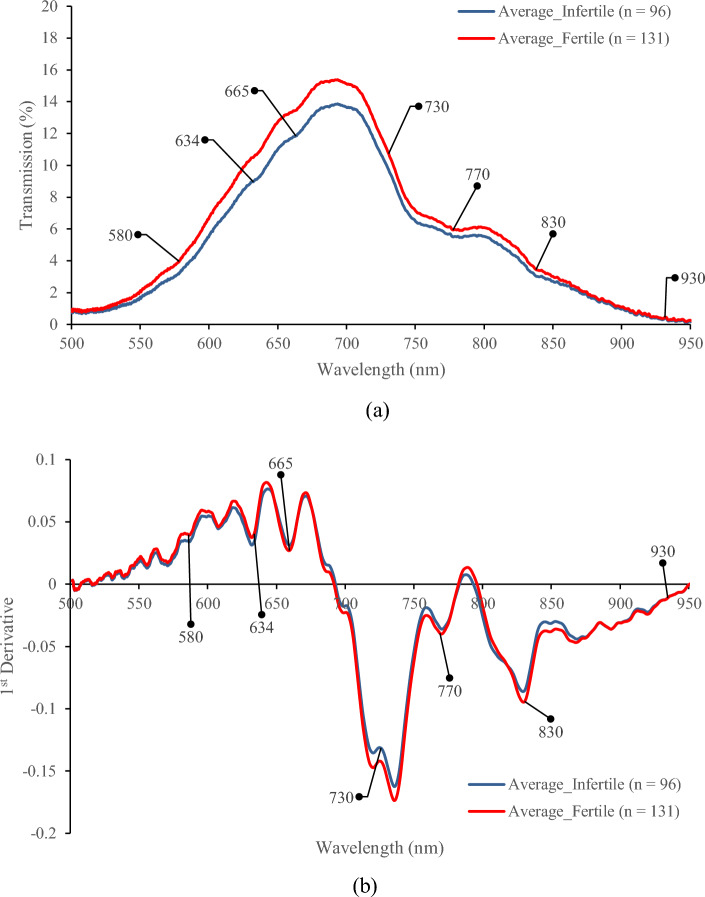
The average transmission spectra of fertile and infertile eggs (**a**) raw spectra and (**b**) 1st derivative spectra.

The raw and 1st derivative of average transmission spectra of fertile and infertile eggs are shown in Fig. 4. As shown, the fertile samples had higher raw transmission values than the infertile ones in all spectral regions (Fig. 4a). Differences in chemical substances, such as protein content^18,19^, and physical properties, like the egg shape index^8^, between fertile and infertile chicken eggs could lead to variations in the transmission spectral behavior. Similar transmission values and trends in the spectra of fertile and infertile eggs were observed in the studies performed by conventional transmission spectroscopy systems^8,15,29^. However, more absorption bands appeared in transmission spectra obtained by the HSI system. As shown in Fig. 4, in addition to broad absorption bands, significant absorptions (valleys in transmission spectra) were revealed in the spectra of fertile and infertile eggs around the wavelengths of 580, 634, 665, 730, 770, 830, and 930 nm. Since the raw spectra had relatively broad absorption bands, to improve the quality of presentation, the 1st derivative spectra were calculated (Fig. 4b).

As shown in Fig. 4, extensive absorption bands in the visible regions were observed around 580, 634, and 665 nm wavelengths. Considering the white shell of eggs used in this study, these absorptions cannot be attributed to the shell color pigment of protoporphyrin which produces relatively strong absorption in brown-shelled egg spectra in the mentioned wavelengths^15,29^. Therefore, these broad absorptions were likely due to the color pigments in the yolk such as carotenoids which are a fat-soluble group of yellow (580 nm), orange (634 nm), and red (665 nm) pigments^30^.

The wavebands around 730 nm are related to the third overtone of O–H in water^31^ and the carbohydrates of eggs. Since the embryo consumes carbohydrates, proteins, and fats^32^, the higher transmission value of fertile samples around 730 nm is likely due to the relatively lower carbohydrate, protein, and fat contents of fertile samples with respect to the infertile ones (Fig. 4a).

The relatively similar internal conditions and common compounds between these two groups of samples could result in similar absorption bands and make it difficult to distinguish the two classes based on the visual interpretation of spectra. However, by a close investigation of 1st derivative spectra (Fig. 4b), the difference in transmitted light was observed between two classes around the discussed wavelengths. The remarkable deviations between the two classes could be observed around the wavelengths of 730, 770, and 830 nm. While the red-edge wavelengths around 770 nm^33^ were related to embryo development^1^, absorption around 830 nm is triggered by the 3rd overtone of the O–H stretching, related to the water content inside of eggs^31^. Finally, the strong absorption around 930 nm can be associated with 3rd overtones of the C-H stretching absorption, which may be related to the carbohydrate content in the egg^34^.

### Classification by SIMCA

Table 1 shows the test set validation results of SIMCA analysis performed by using entire spectral data for the detection of infertile and fertile eggs before incubation. The effect of different pre-processing and the optimum number of PCs for modeling each class were also presented in this table. Among the various mathematical pretreatments, the best discrimination accuracy was achieved from the 1st and 2nd derivatives. In both pretreatments, all fertile eggs were correctly classified (sensitivity of 100%) and 6 infertile eggs were misclassified into fertile (specificity of 68.42%), resulting in an accuracy of 86.67%. Both pretreatments led to a similar precision of 81.25%. Due to the higher price of fertile eggs, it is more important to correctly identify the fertile eggs prior to incubation to avoid unwanted elimination and increase the hatchability rate. Therefore, the best SIMCA model had promising performance in the correct identification of this group of eggs.

**Table 1:** Test set validation results of SIMCA analysis for the detection of infertile and fertile eggs before incubation or day 0.

Preprocess	Class	No. of PCs	Classified into		Sen. (%)	Spe. (%)	Precis. (%)	Acc. (%)
			Infertile	Fertile				
Raw	Infertile (n = 19)	1	8	11	100.00	42.11	70.27	75.56
	Fertile (n = 26)	1	0	26				
Norm	Infertile (n = 19)	4	12	7	96.15	63.16	78.13	82.22
	Fertile (n = 26)	4	1	25				
MSC	Infertile (n = 19)	4	12	7	96.15	63.16	78.13	82.22
	Fertile (n = 26)	4	1	25				
SNV	Infertile (n = 19)	4	12	7	92.31	63.16	77.42	80.00
	Fertile (n = 26)	5	2	24				
BC	Infertile (n = 19)	1	9	10	100.00	47.37	72.22	77.78
	Fertile (n = 26)	1	0	26				
1st Der	Infertile (n = 19)	4	13	6	100.00	68.42	81.25	86.67
	Fertile (n = 26)	4	0	26				
2nd Der	Infertile (n = 19)	8	13	6	100.00	68.42	81.25	86.67
	Fertile (n = 26)	5	0	26				

1st Der.: First derivative; 2nd Der.: Second derivative; Acc.: Accuracy; BC: Baseline correction; MSC: Multiplicative scatter correction; Norm.: Normalization; PCs: Principal components; Percis.: Precision; Preprocess.: Preprocessing; Sen.: Sensitivity; SNV: Standard normal variate; Spe.: Specificity.

In terms of fertile and infertile detection of eggs before incubation by HSI, our predictions based on the SIMCA methods were much better than those reported by Smith, et al.^13^ (accuracy of 63% for day 0, before incubation), Lin, et al.^6^ (accuracy of 96% for day 14, after incubation), and Park, et al.^1^ (accuracy of 99% for day 14, after incubation). In one case, however, our best SIMCA model was less accurate than that presented by Liu and Ngadi (2013) in which a near-infrared HSI system in the range of 900–1700 nm was used for the detection of fertile and infertile eggs prior to incubation. The model extracted by them was able to correctly classify all fertile and infertile eggs into the right classes with an overall accuracy of 100%. Despite the same performance in the detection of fertile eggs (100%), our best SIMCA model was weaker in the detection of infertile ones (specificity of 68.42%). The limited waveband (430–960 nm) in our study provided by a lower-price hyperspectral camera can be regarded as one of the main reasons for obtaining these results. Nevertheless, in the study of Liu and Ngadi (2013), there was an unequal distribution of samples per class on day 0 (prior to incubation), where a total of 18 infertile eggs were used against 156 fertile ones in the training and validating datasets. There was no justification for solving the imbalanced classification problems. The main challenge here was how much the use of advanced multivariate techniques could compensate the drawback of the shorter spectral range.

**Figure 5:**
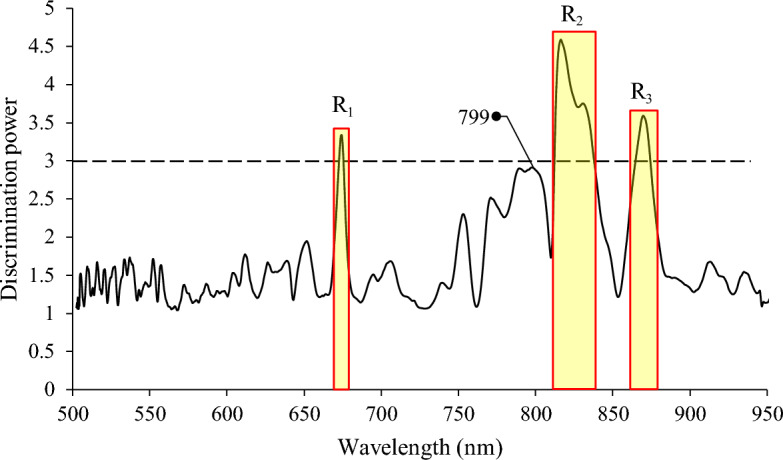
The discrimination power plot of wavelength variables obtained by 1st derivative pretreatment and projecting the data from the fertile model onto the infertile ones.

Figure 5 shows the discrimination power plot of different wavelengths for the separation of the fertile (day 0) class from the infertile one obtained from the best model by 1st derivative pretreatment. This plot shows which wavelengths were most effective for distinguishing between two classes. As a rule, variables that resulted in a discrimination power of more than 3 are very useful in distinguishing between two classes^22^. These variables were specified by distinct regions and relatively narrow bands in Fig. 5, represented by R_1_ (673–675 nm), R_2_ (813–840 nm), and R_3_ (865–873 nm). Additionally, the discrimination power values around the wavelengths of 799 nm were close to 3, indicating the relative importance of this wavelength. It is noteworthy that important areas were located mostly at the end of the visible region (R_1_) and NIR region (799 nm, R_2_, and R_3_). The effect of fertility on wavelengths around 799 nm can be related to the possible formation of blood spots due to the initial stages of embryo development. By examining the PCA images, Park, et al.^1^ observed a distinct blood vessel pattern in the viable eggs and reported a height weighting coefficient value of around 799 nm in the corresponding PCA loading plot.

Moreover, it seems that the presence of the embryo could influence the NIR region more significantly than the visible one (Fig. 5). It can be due to the possible changes in the egg’s chemical composition because of embryo existence^17,30,35^, making the NIR region more important in separating fertile eggs from infertile ones. The initial parts of the spectrum, especially the wavelength range of 500–650 nm had a lower degree of importance for separation between the two classes.

Among the distinguished regions, the R_2_ region, including the important wavelength of 830 nm, resulted in the highest discrimination power values (Fig. 5). By referring to Fig. 4, the wavelengths around 830 nm provided a relatively strong absorption for both fertile and infertile eggs with the remarkable absorption difference between these two groups of samples. As indicated in the overview of spectral data section, the absorptions around 830 nm can be related to 3rd overtones of the O–H stretching absorption, which according to egg ingredients can be attributed to the carbohydrates and water content of the egg. Since the embryo consumes carbohydrates, proteins, and fats during its development^32^, the higher discrimination power values of the R_2_ region were likely due to the relatively lower carbohydrate, protein, and fat contents of fertile samples with respect to the infertile ones.

### Discrimination by LDA and QDA

Table 2 summarizes the test set validation results of QDA and LDA classifiers obtained by various mathematical pretreatments. Similar to SIMCA analysis, the best discrimination accuracy was achieved by the 1st and 2nd derivatives. In the best QDA and LDA models, all of the fertile eggs were correctly classified (sensitivity of 100%) and 5 infertile eggs were misclassified into fertile (specificity of 73.68%), resulting in an accuracy of 88.90%. The maximum precision of both models reached 83.87%. In comparison with the SIMCA analysis with the accuracy and precision of 86.67% and 81.25% respectively, the best QDA and LDA models resulted in slightly better performance (accuracy of 88.90% and precision of 83.87%), indicating 2.6% and 3.2% improvement in discrimination accuracy and precision, respectively. The relatively similar performance of the LDA and QDA methods, when compared to the SIMCA method, suggested that more advanced approaches, capable of better capturing the potential nonlinear nature of the data, are necessary to develop more robust predictive models.

**Table 2:** Test set validation results obtained by LDA and QDA analysis for the detection of infertile and fertile eggs before incubation or day 0.

Classifier	Preprocess	Sen. (%)	Spe. (%)	Precis. (%)	Acc. (%)
LDA	Raw	92.31	68.42	80.00	82.22
	Normalized	88.46	68.42	79.31	80.00
	MSC	92.31	68.42	80.00	82.22
	SNV	92.31	68.42	80.00	82.22
	BC	76.92	73.68	80.00	75.56
	1st Der	100.00	73.68	83.87	88.89
	2nd Der	100.00	73.68	83.87	88.89
QDA	Raw	80.79	73.68	80.77	77.78
	Normalized	73.1	73.68	79.16	73.33
	MSC	80.77	68.42	77.78	75.56
	SNV	80.77	68.42	77.78	75.56
	BC	84.62	73.68	81.48	80.00
	1st Der	100.00	73.68	83.87	88.89
	2nd Der	100.00	73.68	83.87	88.89

1st Der.: First derivative; 2nd Der.: Second derivative; Acc.: Accuracy; BC: Baseline correction; LDA: Linear discriminant analysis; MSC: Multiplicative scatter correction; Norm.: Normalization; PCs: Principal components; Percis.: Precision; Preprocess.: Preprocessing; QDA: Quadratic discriminant analysis; Sen.: Sensitivity; SNV: Standard normal variate; Spe.: Specificity.

### Linear classification by effective wavelengths

Table 3 summarizes the results of linear classifiers, including SIMCA, LDA, and QDA, for fertility detection using the selected regions (R_1_, R_2_, and R_3_) and the spectral differences between two pairs of wavelengths in these selected regions as input variables. As shown, employing the effective wavelengths in the selected regions did not yield promising performances across all classifiers. In the best-case scenario, SIMCA achieved a sensitivity of 61.54%, specificity of 73.68%, precision of 76.19%, and accuracy of 66.67%. It appears that the wavelength variables situated outside the selected region contained some information to describe the variance of the class variable (fertile or infertile), essential for developing a reliable linear model. Furthermore, the utilization of selected variables in their raw form, combined with their inclusion in linear models lacking the capacity to address nonlinear data, contributes to the challenges encountered in fertility detection using selected regions.

**Table 3:** Linear classification results using effective wavelengths in selected regions and spectral differences between two pairs of wavelengths in selected regions for detection of infertile and fertile eggs on day 0.

Classifier	Input variables	Class	Classified to		Sen. (%)	Spe. (%)	Percis. (%)	Acc. (%)
			Infertile	Fertile				
SIMCA	R_1_ + R_2_ + R_3_	Infertile (n = 19)	14	5	61.54	73.68	76.19	66.67
		Fertile (n = 26)	10	16				
	Spectral difference	Infertile (n = 19)	16	3	61.54	84.21	84.21	71.11
		Fertile (n = 26)	10	16				
LDA	R_1_ + R_2_ + R_3_	Infertile (n = 19)	14	5	53.85	73.68	73.68	62.22
		Fertile (n = 26)	12	14				
	Spectral difference	Infertile (n = 19)	17	2	80.77	89.47	91.30	84.44
		Fertile (n = 26)	5	21				
QDA	R_1_ + R_2_ + R_3_	Infertile (n = 19)	14	5	53.85	73.68	73.68	62.22
		Fertile (n = 26)	12	14				
	Spectral difference	Infertile (n = 19)	15	4	84.62	78.95	84.62	82.22
		Fertile (n = 26)	4	22				

Acc.: Accuracy; LDA: Linear discriminant analysis; Percis.: Precision; QDA: Quadratic discriminant analysis; Sen.: Sensitivity; Spe.: Specificity.

However, when utilizing wavelength differences as a simulation of 1st derivative pretreatment, predictability improved, although it remained lower than the models developed using the entire spectra as the input variable. For SIMCA, LDA, and QDA, accuracy values of 71.11%, 84.44%, and 82.22% were obtained, respectively (Table 3). In comparison, the corresponding values when using the entire spectral data were 86.67%, 88.89%, and 88.89%, respectively (Tables 1 and 2). It seems that spectral differences can effectively simulate the 1st derivative pretreatment in distinct wavelength variables. Nevertheless, linear classification still does not reach the same level of predictability as when the entire spectral data is utilized.

### Classification by ANN

**Figure 6:**
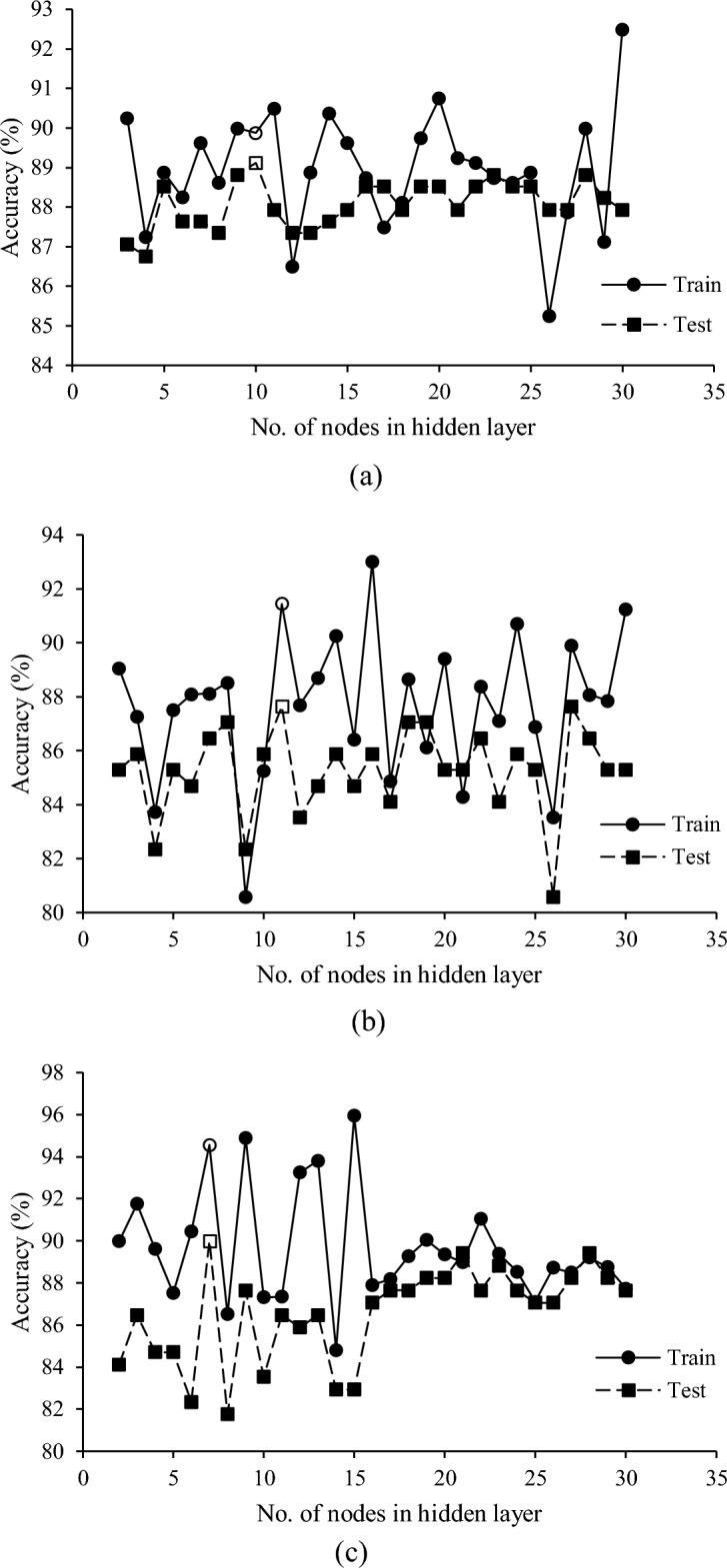
The prediction power of ANN models with a different number of nodes in the hidden layer for detecting fertile and infertile eggs (**a**) total spectra, (**b**) selected regions, and (**c**) spectral difference.

Table 4 shows the results ANN classifier obtained from the total spectral data, effective wavelengths in selected regions (R_1_, R_2_, and R_3_), and spectral differences between two pairs of wavelengths in selected regions, as the input variables. Figure 6 illustrates the classification accuracy of ANNs with the different number of nodes in the hidden layer varying from 1 to 30. In using the total spectral data as the inputs to networks (Fig. 6a), the best prediction accuracy was achieved by 10 nodes in the hidden layer. This network was able to separate the fertile egg from the infertile one with sensitivity, specificity, precision, and accuracy of 96.15%, 84.21%, 89.29%, and 91.11% (Table 4). As shown, a noticeable improvement in the prediction power of infertile eggs occurred when the nonlinear ANN method was applied to the whole spectral data. While the best linear model could distinguish 73.68% of infertile eggs, 84.21% of infertile eggs could be successfully discriminated by the best ANN model based on total spectral data. This showed a 14.3% improvement in the specificity of the predictive model.

**Table 4:** Nonlinear classification results for the detection of infertile and fertile eggs on day 0.

Input variables	ANN topology	Class	Classified to		Sen. (%)	Spe. (%)	Percis. (%)	Acc. (%)
			Infertile	Fertile				
Total spectral data	256–10-2	Infertile (n = 19)	16	3	96.15	84.21	89.29	91.11
		Fertile (n = 26)	1	25				
R_1_ + R_2_ + R_3_	38–11-2	Infertile (n = 19)	17	2	96.15	89.47	92.59	93.33
		Fertile (n = 26)	1	25				
Spectral difference	667–7-2	Infertile (n = 19)	16	3	100.00	84.21	89.65	93.33
		Fertile (n = 26)	0	26				

Acc.: Accuracy; Percis.: Precision; Sen.: Sensitivity; Spe.: Specificity.

**Figure 7:**
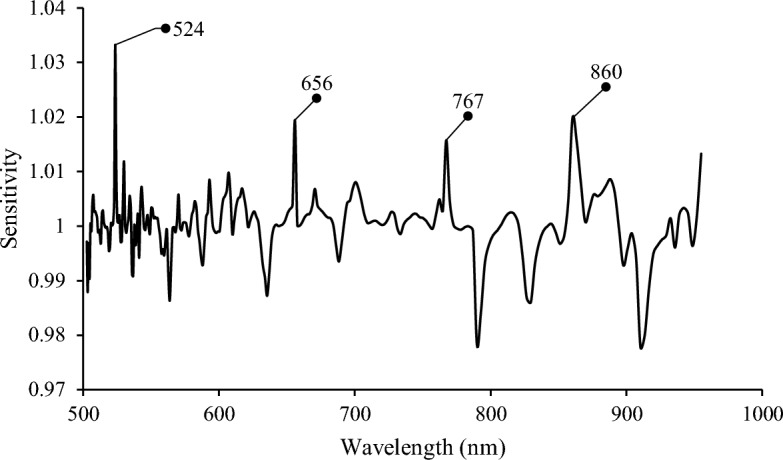
Sensitivity analysis plot based on best ANN model obtained from total spectral data.

Figure 7 illustrates the sensitivity plot based on the best ANN model developed by the total spectral data. As shown, the wavelengths around 524, 656, 767, and 860 nm resulted in the highest degree of importance in the sensitivity analysis. These wavelengths were also close to the distinguished ones that appeared in Fig. 4 as the absorption valleys in transmission spectra. Moreover, the wavelengths around 665 and 865 nm were identified as the efficient wavelengths in the discrimination power plot of SIMCA analysis. The coincidence in the identified wavebands between linear (SIMCA) and nonlinear (ANN) approaches highlighted the importance of the selected regions R_1_ and R_3_ in detecting fertility in unincubated chicken eggs.

In using the selected regions (Fig. 6b) and spectral differences (Fig. 6c) as the input variables to the ANN classifier, the optimized topologies of networks were attained with 11 and 7 nodes in the hidden layer, respectively. Despite a slight difference in precision values attained by selected regions and their spectral differences (92.59% and 89.65%, respectively), similar accuracies (accuracy of 93.33%) were achieved when the selected regions and spectral differences were used as the input variables (Table 4). Interestingly, and in contrast to the linear classifiers, the accuracy of prediction slightly improved when employing fewer variables from effective regions or spectral differences as input for nonlinear ANN methods, compared to using the full range data (accuracy of 91.11%). This improvement may be attributed to the elimination of unnecessary and irrelevant data that could otherwise have a detrimental impact on the ANN models.

Among the best networks, the ANN model developed by the effective region was the most successful one in separating the infertile eggs with a specificity of 89.47%. Only 2 infertile eggs were misclassified as fertile. However, the model developed by spectral differences was more efficient in predicting the fertile eggs in which all of them were correctly classified (sensitivity of 100%).

On the whole, the ANN method outperformed the other classifiers in detecting fertility. Compared to the best SIMCA and LDA/QDA models, the accuracy was improved by 7.7% and 5% when the best ANN model was used to discriminate the eggs.

## Conclusion

In this study, the feasibility of a line-scan hyperspectral imaging system in the Vis-SWNIR region was assessed for early detection of non-fertile eggs on day 0 before incubation. After capturing hyperspectral images of eggs, the edge detection method was used to segment ROI, and the image spectral information was extracted from the ROI. Then various pretreatment methods as well as different classifiers including SIMCA, LDA, QDA, and ANN classifiers were applied to eliminate the unwanted information and extract the predictive models. The following conclusions can be drawn from our results:

1. Following the acceptable results of SIMCA analysis along with 1st derivative pretreatment (accuracy of 86.67%), the discrimination power plot was used to select the most informative wavebands in discerning the fertile and non-fertile classes.
2. The best QDA and LDA models resulted in slightly better performances in comparison with the SIMCA analysis. Surprisingly, all the fertile eggs were correctly classified in these models, resulting in a sensitivity of 100%.
3. The best ANN model based on total spectral data was able to separate the fertile eggs from the infertile ones with precision and accuracy of 89.29% and 91.11%, respectively. Whereas, by using the fewer and most informative wavelengths obtained by SIMCA analysis as the input variable to ANN models, an improvement in prediction power occurred. Both wavelength differences and raw spectral data in selected wavebands led to a satisfactory classification accuracy of 93.33%. None of the infertile eggs were misclassified as fertile in the models based on wavelength differences (a sensitivity of 100%).

## Data Availability

The datasets generated and/or analyzed during the current study are not publicly available, but are available from the corresponding author on reasonable request.
